# Vaccination: Public and Private

**Published:** 1901-11-23

**Authors:** 


### The Hospital

A Journal of

The Medical Sciences and Hospital Administration.

Vol. XXXI.—No. 791.] Edited by Sir Henry Burdett, K.C.B., and Solomon C. Smith, M.D., M.R.C.P. [November 23, 1901.

### Vaccination: Public and Private.

At the present moment, with what threatens to become a big epidemic of small-pox growing daily in our midst, the necessity of vaccination and re-vaccination is sufficiently obvious, as it also is that the operation should be done properly and effectually. Hence, seeing that by Act of Parliament an official scheme of free vaccination has been set up, it appears not unreasonable that some notification should be given to the people as to where and by whom the operation can be properly done. The question which is just now agitating the medical world is by whom should this announcement be made; and, although we do not wish to pose as purists, we cannot but feel that the public vaccinators themselves should be the last people in the world to advertise the goodness of their wares. They, however, seem to think otherwise. Hence the outcry. What has happened is that a statement has appeared in the public press, purporting to have been issued by the organising secretary of the Association of Public Vaccinators, in which attention is directed to the manner in which the names and addresses of public vaccinators can be obtained, and to the fact that "public vaccinators are the only medical men who are compelled to use the safeguards prescribed by the regulations of the Local Government Board, and that they are the only persons who can obtain the pure glycerinated calf lymph prepared in the laboratories of the Local Government Board, and that they are compelled to use that lymph in all cases of vaccination and re-vaccination in their own district."

We can hardly be surprised that professional men who have been brought up in the belief that advertising is wrong, and that to lay claim to any special ability in comparison with one's fellows is absolutely "infamous," as the Medical Act has it, should feel aggrieved at the very open self-assertion contained in this curious document. No one can doubt that its intention (to use the terms employed in the celebrated case of Dr. Irvine) is to "solicit and invite" people to apply to the public vaccinators, by whose directions it is issued, or that it "refers" to the "special ability" of these gentlemen in regard to the performance of vaccination; nor can we doubt that, if any private vaccinator were to advertise his own special abilities in the way in which the special advantages of going to public vaccinators have been advertised in the document to which we have referred, he would run considerable risk of being hauled up before that august body, the General Medical Council. So far then as the question is one of method, of taste, and of professional propriety, we quite agree with those practitioners who have protested in the strongest terms against the proceedings of the public vaccinators as voiced by their Association. All the same, we cannot but suspect that a good deal of their anger is caused, not entirely by the outrage offered to ethical propriety, but partly by the consciousness that the advertisement is true, and that this attempt to drive vaccination into the hands of the public vaccinators is but the natural result of the "one mark" and inefficient vaccination by which the credit of the operation has during recent years been steadily undermined. The "monopoly," so-called, of the public vaccinator is but a rebound from the inefficiency of much private practice vaccination, and evidently the official plan is to draw vaccination as much as possible into official hands.

If it had been possible in arranging the details of the Vaccination Act to enlist the services and secure the co-operation of the whole profession, many difficulties would have been avoided and vaccination would have been greatly popularised, and we can hardly doubt that in deliberately throwing away such great advantages as that course offered, the framers of the Act were influenced by a profound distrust of private practice vaccination. Evidence of the same distrust and the same determination to keep the vaccination of the people as far as possible in the hands of responsible officials is evidenced again in the refusal to supply the Government calf lymph to any but public vaccinators, and the result is that the private vaccinators are left at a serious disadvantage.

One effect of the Vaccination Act has been that calf lymph is now demanded by everyone, and as a result no vaccinator knows, of his own knowledge, anything about the lymph he uses. The supply of the material has become a matter of commerce, and commercial methods do not always give satisfactory guarantees as to the purity and activity of such an article as vaccine lymph. Moreover, the doctor may have "notions" as to the mode of operating, the degree of protection which is requisite, and the kind of antiseptic measures which are desirable. There is no standard, and but little rule. Some private practitioners vaccinate splendidly, some—well, just the opposite ; and each is the sole judge of his own performances. Under these circumstances, can we doubt that if vaccination and re-vaccination are desirable things, it is desirable also that the whole affair should be standardised ; that the lymph should be got, not from a shop, but from a Government laboratory ; that the doctor should be bound by rules to certain standard methods ; and finally, that the public should be officially informed where such standard vaccination can be obtained ? No one, we think, can question this. But we all may question the propriety of the public vaccinators themselves, or through their secretary, undertaking the delicate task of blowing their own trumpets.

